# Body Shape Indices and Chronic Knee Pain in Chinese Aging Population: A Longitudinal Analysis of Abdominal Obesity, BMI, and Waist Circumference

**DOI:** 10.1155/jobe/2379392

**Published:** 2026-09-28

**Authors:** Dizhou Zhao, Daiqiang Huang, Lifen Zhang, Yunhui Wang, Lu Liu, Xiangyan Jin, Zhiming Shan

**Affiliations:** ^1^ Department of Anesthesiology, Shenzhen People’s Hospital, The First Affiliated Hospital, Southern University of Science and Technology, Shenzhen, China, sustc.edu.cn

**Keywords:** abdominal obesity, body shape indices, CHARLS, chronic knee pain

## Abstract

**Background:**

Chronic knee pain (CKP) is a globally prevalent condition among older adults, yet the predictive value of diverse obesity‐related indices for CKP remains insufficiently explored in the Chinese population. This study aims to investigate the longitudinal relationship between abdominal obesity, multiple body shape indices (including weight, body mass index [BMI], waist circumference [WC], visceral adiposity index [VAI], and conicity index [CI]), and the risk of developing CKP using data from the China Health and Retirement Longitudinal Study (CHARLS).

**Methods:**

We conducted a longitudinal analysis using data from CHARLS Wave 3 (2015, baseline) to Wave 5 (2020). The inclusion criteria were participants aged 55 years or older with complete biomarkers from fasting blood samples and anthropometric data at baseline. We excluded participants who: (1) had missing blood test results or body index data, (2) were lost to follow‐up, (3) had missing information regarding knee pain, and (4) reported pre‐existing knee pain at baseline (Wave 3). Abdominal obesity and body shape indices were assessed at baseline. The incidence of CKP was defined as reporting knee pain in two consecutive follow‐up waves. Logistic regression and restricted cubic splines were employed to evaluate the associations.

**Results:**

889 out of 3288 participants, which is 27.04%, were identified as CKP during the 5‐year follow‐up period (2015–2020). After adjusting for various confounding factors, the OR for CKP associated with BMI was 1.03 (95% CI: 1.01–1.05; *p* = 0.020), WC was 1.01 (95% CI: 1.01–1.02; *p* = 0.011), and people with abdominal obesity was 1.36 (95% CI: 1.15–1.60; *p* < 0.001). Subgroup analysis demonstrated similar results without any interactions. In restricted cubic spline regression, the relationship between BMI and CKP is nonlinear, and the relationship between WC and CKP is linear.

**Conclusions:**

Among middle‐aged and older adults, abdominal obesity, BMI, and WC should be regarded as risk factors of CKP, whereas VAI, CI, and body weight should not. Notably, this study revealed a nonlinear relationship between BMI and CKP, contrasting with the linear link observed between WC and CKP. Furthermore, compared to BMI and WC, abdominal obesity demonstrates a higher statistical effect size and more pronounced clinical significance.

## 1. Introduction

Chronic knee pain (CKP) is a prevalent and debilitating condition that significantly impacts the quality of life for many individuals, particularly among the aging population in China. The Chinese prevalence of CKP is estimated at approximately 8.1%, and this figure is climbing [1, 2]. This surge is attributed to multiple factors, including an aging population, increasing rates of obesity, and a growing burden of joint injuries [3, 4].

The management of CKP presents considerable challenges. While advancements in imaging techniques like X‐rays and MRI have improved diagnostic precision, treatment remains complex, often necessitating a combination of pharmacological and non‐pharmacological interventions [5]. The economic and healthcare burden of CKP is substantial, with direct costs associated with medical treatment and indirect costs due to loss of productivity and diminished quality of life [6]. The impact of CKP extends beyond the individual, highlighting the need for public health strategies and economic planning to address this growing issue [7].

The increase in obesity rate might be a significant factor for China’s aging population. According to a study published in 2023, the prevalence of overweight increased from 12.82% in 2008 to 28.45% in 2018, while obesity rates rose from 1.62% to 4.95% during the same period [8, 9]. Analyzing the association between obesity and CKP and developing related public health policies may be a very appropriate strategic direction for China’s aging population.

Additionally, an increasing number of studies suggest that relying solely on BMI is inadequate when considering indicators related to obesity [10]. Abdominal obesity is believed to be significantly and causally linked to a broader spectrum of diseases. Furthermore, it is essential to incorporate visceral fat, blood lipid levels, and similar metrics into the reference indicators of obesity to comprehensively assess the associated health risks [11, 12].

The aim of this study was to investigate the link between obesity and CKP among a cohort of retired Chinese older adults. Transcending the traditional reliance on BMI, this research incorporates a holistic suite of obesity indicators, including abdominal obesity, waist circumference (WC), visceral adiposity index (VAI), and conicity index (CI), to paint a comprehensive picture of how different dimensions of obesity intersect with CKP. This study promises to shed light on the nuanced ways in which obesity may contribute to CKP, offering a foundation for more targeted interventions.

## 2. Methods

### 2.1. Study Design and Population

Our study is anchored in data extracted from the China Health and Retirement Longitudinal Study (CHARLS), a nationally representative longitudinal survey encompassing individuals aged 45 and above in China. The first CHARLS survey was conducted between 2011 and 2012 (Wave 1), followed by four waves of follow‐up investigations (Wave 2 in 2013, Wave 3 in 2015, Wave 4 in 2018, and Wave 5 in 2020). This study used data from the CHARLS database. Ethical approval and informed consent procedures were conducted by the original data providers.

In this study, we recruited 16,406 participants who completed the Biomarker Questionnaire in 2015 (Wave 3) as the observation commencement point. We then excluded 3316 participants for missing blood test results and body index data. Participants who were lost to follow‐up at any following wave (*n* = 1823) and missed the information of knee pain (*n* = 5564) were excluded. Participants for missing gender information and age < 55 were also excluded. Participants who had already claimed knee pain in Wave 3 were also excluded (*n* = 2067). Finally, a total of 3288 participants were recruited for further analysis (shown in Figure 1).

**FIGURE 1:**
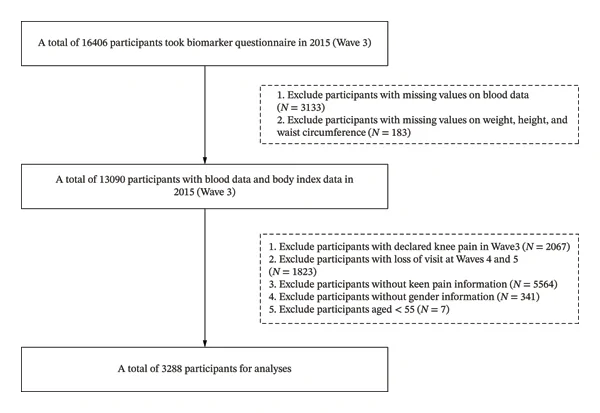
Flowchart of the study population.

### 2.2. Data Collection on Abdominal Obesity and Body Shape Indices

All participants completed the biomarker questionnaire and fasting blood test in the 2015 survey (Wave 3). In Wave 3, participants determined their weight with a scale and their height with a stadiometer, subsequently documenting these measurements in the biomarker questionnaire. The investigator wrapped the soft tape measure around the participant’s umbilicus, instructing them to calm their breath and hold it at the end of an exhalation. The WC, indicated by the tape’s degree marking, was recorded in the biomarker questionnaire. We could collect the information of triglyceride (Tg) and high‐density lipoprotein (HDL) in the blood test database. Defining abdominal obesity and obtaining body shape indices requires the application of the above parameters.

Abdominal obesity: males with WC ≥ 90 cm, females with WC ≥ 80 cm [13, 14].

(1) BMI=WeightHeight2,

(2) Males VAI=WC36.58+1.88×BMI+TG1.03+1.31HDL,

(3) Females VAI=WC36.58+1.89×BMI+TG0.81+1.52HDL,

(4) CI=WCm0.019Weightkg/heightm.

### 2.3. Data Collection on CKP

All participants completed the “Health status and functioning” questionnaire [15] from the CHARLS survey in Waves 3, 4, and 5. And the participants correctly reported pain information by answering the question, “What part of your body do you feel pain? Please list all the parts of your body where you currently feel pain” in each wave. CKP was defined as the new onset of knee pain reported in both 2018 (Wave 4) and 2020 (Wave 5), among participants who were pain‐free at baseline in 2015 (Wave 3).

### 2.4. Data Collection and Ascertainment of Covariates

Demographic variables, such as age, sex, marital status, residential area, smoking and drinking habits, systolic blood pressure (SBP), diastolic blood pressure (DBP), body mass index (BMI), comorbidities (hypertension, dyslipidemia, diabetes, rheumatism, and so on) and history of analgesic use, were all gathered from the baseline questionnaire administered during Wave 3.

### 2.5. Directed Acyclic Graph (DAG) for Covariate

To logically identify potential confounders and clarify the complex causal pathways between obesity‐related indices and CKP, a DAG was constructed (Figure 2). Notably, variables identified as potential mediators on the causal pathway were not adjusted for in the primary models to estimate the total effect of obesity on CKP.

**FIGURE 2:**
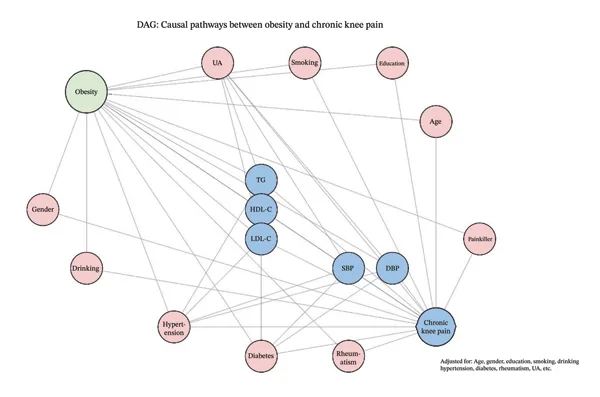
DAG illustrating the assumed causal relationships: Pink points represent variables defined as confounders and were adjusted for in the primary models. Blue points represent variables defined as mediators on the causal pathway and were not adjusted for in the primary model to estimate the total effect. Abbreviations: DBP: diastolic blood pressure; HDL‐C: high‐density lipoprotein cholesterol; SBP: systolic blood pressure; TG: triglyceride.

### 2.6. Statistical Analysis

The data were analyzed using SPSS Version 27.0, STATA/SE 16.0, and R Version 4.3.0. A *p* value of less than 0.05 was used to determine statistical significance. The mean and standard deviation (SD) of continuous data and categorical data were described by frequencies.

The proportions of missing covariate data are detailed in Table S1, with the highest missing rates observed in drinking status (23.45%) and smoking status (8.76%). To maximize the utilization of available data and minimize potential bias, we employed multiple imputation for handling these missing values. Assuming the data were missing at random (MAR), the multiple imputation model included all pertinent variables used in our subsequent analyses (i.e., outcome variable, demographic characteristics, blood pressure, and all body shape indices). A total of 5 imputed datasets were generated. We carefully evaluated the postimputation data distributions and confirmed that they remained highly consistent with the preimputation distributions (Table S2), indicating that the imputation process did not distort the underlying data structure. Pooled results from these 5 datasets were used for the final logistic regression analyses.

Furthermore, to address the potential selection bias arising from the exclusion of participants with missing data, a sensitivity analysis was conducted using inverse probability treatment weighting (IPTW). The probability of inclusion in the final cohort was estimated using a logistic regression model incorporating baseline covariates, and the inverse of these probabilities was applied as weights in the subsequent outcome models to balance the baseline characteristics between the included and excluded populations.

Continuous variables were compared by ANOVA or *t* tests, and categorical variables were compared by the chi‐square test or Fisher’s exact probability test. Univariate binary and multivariate logistic regression were used to assess the associations between abdominal obesity and body shape indices and the risk of CKP. Furthermore, restricted cubic splines were used to explain linear/nonlinear association between body shape indices and CKP.

## 3. Results

### 3.1. Baseline Characteristics of Participants

The baseline characteristics of the 3288 participants are presented in Table 1. Among them, 889 (27.04%) developed CKP during follow‐up. The median age of the study population was 69 years, and 38.72% of the participants were male. Significant differences between participants with and without CKP were observed in age, sex, height, WC, BMI, uric acid (UA), CI, SBP, abdominal obesity, education level, drinking status, smoking status, chronic heart disease, chronic kidney disease, and rheumatism. In contrast, no significant between‐group differences were observed in body weight, Tgs, HDL‐C, LDL‐C, VAI, DBP, residence, marital status, hypertension, dyslipidemia, diabetes, chronic lung disease, liver disease, stroke, or history of analgesic use.

**TABLE 1:** Baseline characteristics of participants.

Variables	Total (*n* = 3288)	Participants without CKP (*n* = 2399)	Participants with CKP (*n* = 889)	Statistic	*p*
Age, years	69.00 (61.00, 75.00)	68.00 (61.00, 75.00)	70.00 (63.00, 76.00)	*Z* = −4.00	**< 0.001**
Gender, *n* (%)				*χ* ^2^ = 22.76	**< 0.001**
Male	1273 (38.72)	988 (41.18)	285 (32.06)		
Female	2015 (61.28)	1411 (58.82)	604 (67.94)		
Height (cm)	157.26 ± 8.28	157.62 ± 8.31	156.27 ± 8.13	*t* = 4.16	**< 0.001**
Weight (kg)	59.37 ± 11.07	59.36 ± 11.04	59.38 ± 11.15	*t* = −0.05	0.958
WC (cm)	85.38 ± 12.50	85.01 ± 12.71	86.38 ± 11.86	*t* = −2.79	**0.005**
BMI (kg/m^2^)	23.95 ± 3.78	23.83 ± 3.71	24.27 ± 3.95	*t* = −2.91	**0.004**
UA (mg/dL)	4.41 ± 1.23	4.42 ± 1.23	4.08 ± 1.15	*t* = 3.52	**< 0.001**
TG (mg/dL)	143.81 ± 91.49	142.95 ± 91.02	146.14 ± 92.78	*t* = −0.89	0.375
HDL‐C (mg/dL)	51.81 ± 11.46	51.68 ± 11.41	52.17 ± 11.60	*t* = −1.07	0.285
LDL‐C (mg/dL)	116.59 ± 35.02	116.57 ± 35.12	117.05 ± 32.80	*t* = −0.17	0.865
VAI	5.24 ± 4.45	5.15 ± 4.36	5.46 ± 4.69	*t* = −1.76	0.078
CI	1.28 ± 0.14	1.27 ± 0.14	1.29 ± 0.12	*t* = −2.67	**0.008**
SBP (mmHg)	126.92 ± 19.76	126.31 ± 19.78	128.56 ± 19.63	*t* = −2.91	**0.004**
DBP (mmHg)	74.89 ± 11.25	74.75 ± 11.27	75.26 ± 11.21	*t* = −1.14	0.253
Abdominal obesity, *n* (%)				*χ* ^2^ = 20.88	**< 0.001**
No	1768 (53.77)	1348 (56.19)	420 (47.24)		
Yes	1520 (46.23)	1051 (43.81)	469 (52.76)		
Residence, *n* (%)				*χ* ^2^ = 0.14	0.712
Rural	2834 (86.19)	2071 (86.33)	763 (85.83)		
Urban	454 (13.81)	328 (13.67)	126 (14.17)		
Marriage status, *n* (%)				*χ* ^2^ = 1.25	0.264
In marriage	2732 (83.09)	2004 (83.53)	728 (81.89)		
Out of marriage	556 (16.91)	395 (16.47)	161 (18.11)		
Education level, *n* (%)				*χ* ^2^ = 7.01	**0.030**
Illiterate	1316 (40.02)	930 (38.77)	386 (43.42)		
Less elementary	1385 (42.12)	1022 (42.60)	363 (40.83)		
High school or higher	587 (17.85)	447 (18.63)	140 (15.75)		
Drinking status, *n* (%)				*χ* ^2^ = 13.30	**0.001**
No	2493 (75.82)	1782 (74.28)	711 (79.98)		
Less than once a month	341 (10.37)	273 (11.38)	68 (7.65)		
More than once a month	454 (13.81)	344 (14.34)	110 (12.37)		
Smoking status, *n* (%)				*χ* ^2^ = 7.60	**0.006**
No	2117 (64.39)	1511 (62.98)	606 (68.17)		
Yes	1171 (35.61)	888 (37.02)	283 (31.83)		
Hypertension				*χ* ^2^ = 2.78	0.095
No	2533 (77.04)	1866 (77.78)	667 (75.03)		
Yes	755 (22.96)	533 (22.22)	222 (24.97)		
Dyslipidemia				*χ* ^2^ = 0.00	0.955
No	2935 (89.26)	2141 (89.25)	794 (89.31)		
Yes	353 (10.74)	258 (10.75)	95 (10.69)		
Diabetes or high blood sugar				*χ* ^2^ = 1.08	0.298
No	3087 (93.89)	2246 (93.62)	841 (94.60)		
Yes	201 (6.11)	153 (6.38)	48 (5.40)		
Chronic lung diseases				*χ* ^2^ = 1.30	0.254
No	2933 (89.20)	2149 (89.58)	784 (88.19)		
Yes	355 (10.80)	250 (10.42)	105 (11.81)		
Liver disease				*χ* ^2^ = 0.39	0.533
No	3109 (94.56)	2272 (94.71)	837 (94.15)		
Yes	179 (5.44)	127 (5.29)	52 (5.85)		
Chronic heart diseases				*χ* ^2^ = 7.84	**0.005**
No	2876 (87.47)	2122 (88.45)	754 (84.81)		
Yes	412 (12.53)	277 (11.55)	135 (15.19)		
Stroke				*χ* ^2^ = 1.44	0.231
No	3216 (97.81)	2342 (97.62)	874 (98.31)		
Yes	72 (2.19)	57 (2.38)	15 (1.69)		
Chronic kidney disease				*χ* ^2^ = 4.66	**0.031**
No	3052 (92.82)	2241 (93.41)	811 (91.23)		
Yes	236 (7.18)	158 (6.59)	78 (8.77)		
Rheumatism				*χ* ^2^ = 125.04	**< 0.001**
No	1994 (60.64)	1594 (66.44)	400 (44.99)		
Yes	1294 (39.36)	805 (33.56)	489 (55.01)		
History of analgesic use				*χ* ^2^ = 0.03	0.853
No	2725 (82.88)	1990 (82.95)	735 (82.68)		
Yes	563 (17.12)	409 (17.05)	154 (17.32)		

*Note:* Continuous variables were expressed as mean ± standard deviation (SD) in case of normal distribution and compared between two groups by *t*‐test or Mann–Whitney test. If the count variable had a theoretical number < 10, Fisher’s exact probability test was used. Categorical variables are presented as counts (percentages) and compared by the chi‐square test. TG, triglyceride; HDL‐C, high‐density lipoprotein cholesterol. The bold values were used to indicate statistical significance (*p* < 0.05).

Abbreviations: BMI, body mass index; CI, conicity index; CKP, chronic knee pain; DBP, diastolic blood pressure; SBP, systolic blood pressure; VAI, visceral fat index; WC, waist circumference.

Notably, there is a significant difference in the prevalence of abdominal obesity between the two groups, and apart from body weight, CI, and VAI, other body shape indices show variations between the groups, which inspired a further analysis.

Furthermore, we offered a baseline overview of the missing variables prior to interpolation, mirroring the results presented above (Table S2).

### 3.2. Associations Between Abdominal Obesity and Body Shape Indices and CKP

In order to explore the link between abdominal obesity and body shape indices with the risk of CKP, we developed three covariate‐adjusted models for logistic regression analysis, as detailed in Table 2. According to Model II logistic regression, which was adjusted for all covariates from DAG, the ORs (95% CIs) for CKP were 1.03 (1.01–1.05) in BMI with *p* values = 0.020 and 1.01 (1.0–1.02) in WC with *p* values = 0.011. ORs (95% CIs) for CKP in participants with abdominal obesity were 1.36 (1.15–1.60) with *p* values < 0.001. Other indices did not exhibit significant correlations with CKP in Model II. After full adjustment, BMI, WC, and abdominal obesity remained significantly associated with incident CKP, whereas weight, VAI, and CI were not.

**TABLE 2:** Odds ratios for incident chronic knee pain occur in different logistic regression models with obesity‐related indices.

Variables	Crude					Model I					Model II				
	*β*	SE	*Z*	*p*	OR (95% CI)	*β*	SE	*Z*	*p*	OR (95% CI)	*β*	SE	*Z*	*p*	OR (95% CI)
Weight	0	0	0.05	0.958	1.00 (0.99–1.01)	0.01	0	2.43	**0.015**	1.01 (1.01–1.02)	0.01	0	1.95	0.051	1.01 (1.00–1.02)
BMI	0.03	0.01	2.98	**0.003**	1.03 (1.01–1.05)	0.03	0.01	2.96	**0.003**	1.03 (1.01–1.05)	0.03	0.01	2.43	**0.020**	1.03 (1.01–1.05)
WC	0.01	0	2.79	**0.005**	1.01 (1.01–1.02)	0.01	0	2.83	**0.005**	1.01 (1.01–1.02)	0.01	0	2.53	**0.011**	1.01 (1.01–1.02)
VAI	0.01	0.01	1.76	0.079	1.02 (1.00–1.03)	0.01	0.01	0.8	0.421	1.01 (0.99–1.02)	0.01	0.01	0.63	0.527	1.01 (0.99–1.02)
CI	0.83	0.32	2.64	**0.008**	2.30 (1.24–4.27)	0.54	0.3	1.77	0.077	1.71 (0.94–3.11)	0.51	0.31	1.63	0.104	1.66 (0.90–3.05)
Abdominal obesity															
No					1.00 (Reference)					1.00 (Reference)					1.00 (Reference)
Yes	0.36	0.08	4.56	**< 0.001**	1.43 (1.23–1.67)	0.3	0.08	3.76	**< 0.001**	1.35 (1.16–1.59)	0.30	0.09	3.58	**< 0.001**	1.36 (1.15–1.60)

*Note:* Model I, adjusted for age and gender. Model II, adjusted for age, gender, education, smoking status, drinking status, hypertension, diabetes, rheumatism, UA, and history of analgesic use. The bold values were used to indicate statistical significance (*p* < 0.05).

Abbreviations: 95% CI: 95% confidence interval; BMI: body mass index; CI: conicity index; SE: standard error; VAI: visceral adiposity index; WC: waist circumference.

The result indicated that BMI, WC, and abdominal obesity were significantly associated with CKP.

### 3.3. Subgroup Analysis

To delve deeper into the relationship between abdominal obesity and body shape and CKP across various demographic groups, we conducted subgroup analysis. The results of these analyses are presented in Tables 3 and 4. The results showed no interaction between groups. Similar results were observed across the female, rural, marital status, partial height, partial age, people with rheumatism, people without hypertension, dyslipidemia, or diabetes, and people without analgesic use subgroups.

**TABLE 3:** Subgroup analysis of the associations between abdominal obesity and chronic knee pain incidence.

Variables	*n* (%)	Abdominal obesity		OR (95% CI)	*p*	
		No	Yes		*p* for interaction	
All patients	3288 (100.00)	420/1768	469/1520	1.43 (1.23–1.67)	< 0.001	
Gender, *n* (%)						0.095
Male	1273 (38.72)	184/845	101/428	1.11 (0.84–1.46)	0.461	
Female	2015 (61.28)	236/923	368/1092	1.48 (1.22–1.80)	**< 0.001**	
Residence, *n* (%)						0.236
Rural	2834 (86.19)	370/1578	393/1256	1.49 (1.26–1.76)	**< 0.001**	
Urban	454 (13.81)	50/190	76/264	1.13 (0.74–1.72)	0.562	
Marriage status, *n* (%)						0.945
In marriage	2732 (83.09)	348/1483	380/1249	1.43 (1.20–1.69)	**< 0.001**	
Out of marriage	556 (16.91)	72/285	89/271	1.45 (1.00–2.09)	**0.049**	
Height (cm)						0.649
< 151.30	812 (24.70)	130/456	132/356	1.48 (1.10–1.99)	**0.010**	
151.30–156.60	823 (25.03)	105/406	131/417	1.31 (0.97–1.78)	0.079	
156.60–162.80	825 (25.09)	92/450	113/375	1.68 (1.22–2.31)	**0.001**	
> 162.80	828 (25.18)	93/456	93/372	1.30 (0.94–1.80)	0.115	
Age						0.669
< 61	686 (20.86)	73/373	77/313	1.34 (0.93–1.93)	0.113	
61–69	905 (27.52)	97/461	135/444	1.64 (1.21–2.22)	**0.001**	
69–75	786 (23.91)	112/418	117/368	1.27 (0.94–1.73)	0.124	
> 75	911 (27.71)	138/516	140/395	1.50 (1.13–2.00)	**0.005**	
Hypertension						0.025
No	2533 (77.04)	336/1484	331/1049	1.57 (1.26–1.96)	**< 0.001**	
Yes	755 (22.96)	84/284	138/471	0.97 (0.67–1.41)	0.868	
Dyslipidemia						0.823
No	2935 (89.26)	396/1657	398/1278	1.40 (1.15–1.70)	**< 0.001**	
Yes	353 (10.74)	24/111	71/242	1.51 (0.83–2.74)	0.173	
Diabetes or high blood sugar						0.251
No	3087 (93.89)	404/1700	437/1387	1.47 (1.21–1.78)	**< 0.001**	
Yes	201 (6.11)	16/68	32/133	0.85 (0.39–1.86)	0.686	
Rheumatism						0.174
No	1994 (60.64)	193/1077	207/917	1.19 (0.91–1.57)	0.206	
Yes	1294 (39.36)	227/691	262/603	1.61 (1.24–2.08)	**< 0.001**	
History of analgesic use						0.971
No	2725 (82.88)	347/1470	388/1255	1.39 (1.13–1.71)	**0.002**	
Yes	563 (17.12)	73/298	81/265	1.45 (0.94–2.23)	0.090	

*Note:* The bold values were used to indicate statistical significance (*p* < 0.05).

Abbreviations: 95% CI, 95% confidence interval; CKP, chronic knee pain; OR, odds ratio.

**TABLE 4:** Subgroup analysis of the associations between obesity‐related indices and chronic knee pain incidence.

Variables	*n* (%)	CKP incidence (%)	BMI		WC		VAI		CI	
			OR (95% CI)	*p* value	OR (95% CI)	*p* value	OR (95% CI)	*p* value	OR (95% CI)	*p* value
Gender, *n* (%)										
Male	1273 (38.72)	285/1273 (22.39)	0.99 (0.96–1.03)	0.762	1.00 (0.99–1.01)	0.666	1.00 (0.96–1.04)	0.972	1.71 (0.58–5.05)	0.330
Female	2015 (61.28)	604/2015 (29.98)	1.04 (1.01–1.06)	**0.003**	1.01 (1.00–1.02)	**0.002**	1.01 (0.99–1.03)	0.406	2.16 (1.37–4.49)	**0.040**
Residence, *n* (%)										
Rural	2834 (86.19)	763/2834 (26.92)	1.04 (1.02–1.06)	**0.001**	1.01 (1.00–1.02)	**0.003**	1.02 (1.00–1.04)	**0.016**	2.35 (1.23–4.47)	**0.009**
Urban	454 (13.81)	126/454 (27.75)	0.98 (0.93–1.04)	0.538	1.00 (0.98–1.02)	0.851	0.96 (0.91–1.01)	0.159	1.70 (0.17–17.02)	0.653
Marriage status, *n* (%)										
In marriage	2732 (83.09)	728/2732 (26.65)	1.03 (1.01–1.06)	**0.002**	1.01 (1.00–1.02)	**0.005**	1.02 (1.00–1.04)	0.085	2.44 (1.21–4.92)	**0.013**
Out of marriage	556 (16.91)	161/556 (28.96)	1.02 (0.96–1.06)	0.640	1.00 (0.99–1.02)	0.517	1.00 (0.97–1.05)	0.632	1.73 (0.47–6.40)	0.413
Height (cm)										
< 151.30	812 (24.70)	262/812 (32.27)	1.04 (1.01–1.08)	**0.019**	1.02 (1.00–1.03)	**0.014**	1.02 (0.99–1.05)	0.237	1.87 (0.71–4.95)	0.205
151.30–156.60	823 (25.03)	236/823 (28.68)	1.01 (0.97–1.05)	0.594	1.00 (0.99–1.02)	0.476	1.00 (0.97–1.03)	0.823	1.40 (0.46–4.29)	0.558
156.60–162.80	825 (25.09)	205/825 (24.85)	1.06 (1.02–1.10)	**0.008**	1.02 (1.00–1.04)	**0.003**	1.01 (0.98–1.05)	0.496	5.08 (1.10–23.36)	**0.037**
> 162.80	828 (25.18)	186/828 (22.46)	1.00 (0.95–1.05)	0.997	1.01 (0.99–1.02)	0.496	1.01 (0.97–1.05)	0.568	2.49 (0.54–11.50)	0.242
Age										
< 61	686 (20.86)	150/686 (21.87)	1.05 (1.00–1.11)	0.054	1.02 (1.00–1.03)	**0.044**	1.03 (0.99–1.07)	0.100	3.74 (0.73–19.25)	0.114
61–69	905 (27.52)	232/905 (25.64)	1.06 (1.01–1.10)	**0.009**	1.02 (1.00–1.03)	**0.039**	1.02 (0.98–1.05)	0.369	3.31 (0.62–17.82)	0.163
69–75	786 (23.91)	229/786 (29.13)	1.02 (0.98–1.06)	0.276	1.01 (0.99–1.02)	0.351	1.00 (0.97–1.03)	0.928	1.64 (0.57–4.73)	0.364
> 75	911 (27.71)	278/911 (30.52)	1.03 (0.99–1.07)	0.121	1.01 (0.99–1.02)	0.315	1.02 (0.99–1.05)	0.227	1.49 (0.57–3.89)	0.411
Hypertension										
No	2533 (77.04)	667/2533 (26.33)	1.05 (1.02–1.08)	**0.002**	1.02 (1.01–1.03)	**< 0.001**	1.00 (0.97–1.02)	0.836	3.17 (1.25–8.02)	**0.015**
Yes	755 (22.96)	222/755 (29.40)	1.02 (0.97–1.06)	0.457	0.99 (0.98–1.01)	0.452	1.01 (0.97–1.04)	0.688	0.45 (0.13–1.58)	0.214
Dyslipidemia										
No	2935 (89.26)	794/2935 (27.05)	1.04 (1.01–1.06)	**0.005**	1.01 (1.00–1.02)	**0.003**	1.00 (0.98–1.03)	0.743	2.24 (1.03–4.87)	**0.041**
Yes	353 (10.74)	95/353 (26.91)	1.06 (0.99–1.13)	0.114	1.00 (0.97–1.02)	0.816	0.99 (0.93–1.05)	0.694	0.06 (0.00–0.91)	**0.043**
Diabetes or high blood sugar										
No	3087 (93.89)	841/3087 (27.24)	1.04 (1.01–1.06)	**0.005**	1.01 (1.00–1.02)	**0.002**	1.00 (0.98–1.02)	0.783	2.41 (1.09–5.30)	0.029
Yes	201 (6.11)	48/201 (23.88)	1.09 (1.00–1.20)	0.062	0.99 (0.97–1.02)	0.536	1.00 (0.95–1.06)	0.894	0.11 (0.01–1.29)	0.079
Rheumatism										
No	1994 (60.64)	400/1994 (20.06)	1.02 (0.98–1.06)	0.292	1.00 (0.99–1.02)	0.425	0.98 (0.95–1.01)	0.251	1.41 (0.45–4.40)	0.552
Yes	1294 (39.36)	489/1294 (37.79)	1.04 (1.01–1.08)	**0.007**	1.02 (1.01–1.03)	**0.004**	1.02 (0.99–1.05)	0.158	2.34 (0.89–6.12)	0.084
History of analgesic use										
No	2725 (82.88)	735/2725 (26.97)	1.03 (1.00–1.06)	0.034	1.01 (1.00–1.02)	**0.040**	1.01 (0.98–1.03)	0.665	1.76 (0.79–3.91)	0.168
Yes	563 (17.12)	154/563 (27.35)	1.07 (1.02–1.13)	**0.007**	1.02 (1.00–1.04)	**0.048**	0.99 (0.95–1.04)	0.727	1.68 (0.29–9.85)	0.564

*Note:* The bold values were used to indicate statistical significance (*p* < 0.05).

Abbreviations: 95% CI: 95% confidence interval; BMI: body mass index; CI: conicity index; CKP: chronic knee pain; OR: odds ratio; VAI: visceral adiposity index; WC: waist circumference.

### 3.4. Sensitivity Analysis for Selection Bias

To evaluate the impact of excluding participants with missing data, we performed an IPTW sensitivity analysis (Table S3). Following IPTW adjustment, the associations between the targeted obesity indices and CKP remained exceptionally robust. Abdominal obesity, WC, and BMI continued to be significant independent risk factors for incident CKP, with weighted ORs (95% CIs) of 1.35 (1.16–1.58), 1.03 (1.01–1.05), and 1.01 (1.00–1.02), respectively. The effect sizes exhibited minimal attenuation compared to the primary Model II. Consistent with our primary models, VAI and CI showed no significant associations. This indicates that selection bias did not materially alter the primary conclusions of our study.

### 3.5. Restricted Cubic Spline Regression Model

For BMI and WC, which were identified as significantly associated with CKP based on the aforementioned findings, we conducted further analysis using restricted cubic spline regression (as depicted in Figures 3 and 4). The analysis revealed a nonlinear relationship between BMI and CKP, with a *p* value for nonlinearity of 0.017. In contrast, the relationship between WC and CKP was found to be linear, with a *p* value for nonlinearity of 0.356.

**FIGURE 3:**
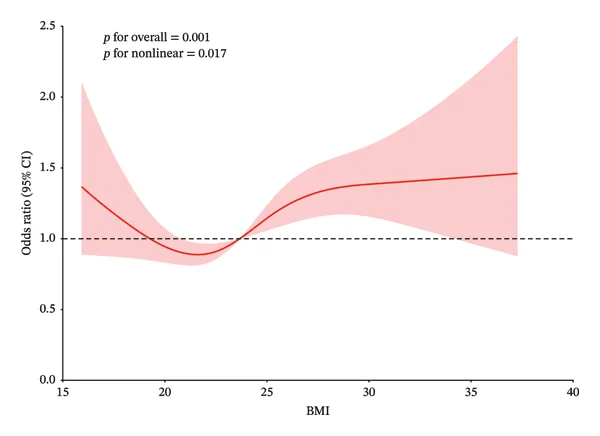
Cubic model of the association between BMI and CKP after adjusting for age, gender, education, smoking status, drinking status, hypertension, diabetes, rheumatism, UA, and history of analgesic use.

**FIGURE 4:**
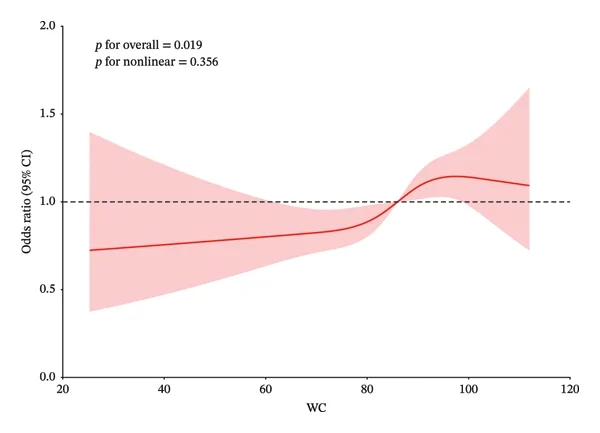
Cubic model of the association between WC and CKP after adjusting for age, gender, education, smoking status, drinking status, hypertension, diabetes, rheumatism, UA, and history of analgesic use.

## 4. Discussion

In China, CKP has seen a notable rise in prevalence and trend. The CHARLS reports that from 2011 to 2018, there was a marked increase in the incidence of CKP among the middle‐aged and older adults in China, and the incidence escalated with advancing age [16, 17]. Obesity is considered to be an important risk factor for the development of CKP. However, relying solely on body weight or BMI to craft public health strategies for CKP has been deemed impractical by numerous studies [18–20]. This is attributed to the intricate mechanisms through which obesity contributes to CKP. Beyond the increased mechanical stress on the joints, chronic inflammation stemming from excess adiposity plays a significant role in obesity‐related CKP [21]. Consequently, this study based on CHARLS endeavored to assess the correlation between body shape indices, abdominal obesity—referring to the accumulation of fat in the abdominal region—and CKP. The aim was to identify more effective predictors to guide the development of CKP prevention policies. To our knowledge, this national retrospective cohort study is the first of its kind to explore the relationship between body shape indices—including weight, BMI, WC, VAI, and CI—and abdominal obesity with respect to CKP among Chinese older individuals.

Participants who denied knee pain in Wave 3 without any missing information were included in the study as the observation starting point. Out of the 3288 participants, a total of 889 consecutively reported knee pain across the five‐year follow‐up encompassing two subsequent waves, leading us to consider them as having CKP. After stratifying the participants based on the presence or absence of CKP, we initially observed that there was almost no difference in body weight between the two groups. And there was a difference in BMI, WC, CI, and abdominal obesity rates. This indicates that it is indeed not feasible to predict the occurrence of CKP solely through screening for body weight.

Further, we constructed a series of logistic regression models to delve into the relationship between body shape indices and abdominal obesity with the occurrence of CKP. In the unadjusted crude model, which did not account for any confounding variables, a high BMI, WC, CI, and the presence of abdominal obesity were identified as independent risk factors for CKP. This finding is consistent with the outcomes of the difference‐in‐differences test. Even after adjusting for various confounders in Model II, BMI (*p* = 0.020, OR (95% CI) 1.03 (1.01–1.05)), WC (*p* = 0.011, OR (95% CI) 1.01 (1.01–1.02)), and the presence of abdominal obesity (*p* < 0.001, OR (95% CI) 1.36 (1.15–1.60) refer to without abdominal obesity) continued to exhibit a significant association with the development of CKP. These indicators should be comprehensively assessed during public health screenings to identify individuals at higher risk for CKP. However, it is crucial to note that while statistically significant, the clinical magnitude of the risk associated with BMI and WC is relatively modest compared to the robust and substantial risk conferred by abdominal obesity.

In interpreting our results, it is important to distinguish between statistical significance and clinical relevance, particularly given our large cohort (*N* = 3288). Although the large sample size may produce small *p* values, the effect sizes observed remain clinically pertinent. The 36% higher risk of CKP associated with abdominal obesity (OR = 1.36) represents a significant burden for older adults.

In particular, we specifically examined the influence of rheumatologic status, as it is a major determinant of knee pain. The positive association between abdominal obesity, BMI, WC, and incident CKP was observed in participants with rheumatism. Meanwhile, no significant interactions were detected between any of the obesity‐related indices and rheumatologic status. This suggests that the impact of adiposity on CKP is robust and pervasive, regardless of underlying organic rheumatologic conditions.

Other subgroup analyses yielded similar results across various groups. This consistency across different subgroups underscores the generalizability of our conclusions regarding the association between BMI, WC, abdominal obesity, and the risk of developing CKP.

Moving forward, we grapple with the question of which among BMI, WC, and abdominal obesity serves as the most optimal risk predictor for CKP. We constructed a restricted cubic spline regression model for BMI and WC and showed a nonlinear relationship for BMI, whereas WC was linear. While the general link between obesity and CKP is well‐documented, the use of a longitudinal incident cohort—excluding baseline cases—coupled with this nonlinear methodological exploration, offers nuanced insights into the different trajectories of these traditional indices. This suggests that in populations with a generally high baseline BMI, the correlation between BMI and CKP might be diminished, whereas WC could be more universally applicable across the entire population. Individuals with a large WC or abdominal obesity may accumulate more fat, particularly visceral fat, compared to those with a BMI. This excess fat storage could potentially intensify the level of chronic inflammation associated with obesity within the body. It bears emphasizing that abdominal obesity has been consistently linked to a myriad of chronic diseases in numerous studies, highlighting its significant health implications [22, 23].

Consequently, we recommend that when conducting screenings for obesity‐related CKP, there should be an increased focus on WC and abdominal obesity alongside BMI. This comprehensive approach will enhance the accuracy of identifying individuals at risk for CKP.

There were also some limitations of the study. While the fundamental association between obesity and CKP has been extensively reported, this study was specifically designed to provide a more granular dissection of this relationship. We acknowledge that our focus on traditional body shape indices, rather than emerging biomarkers, may to some extent constrain the perceived groundbreaking innovation of the work. However, the longitudinal framework of our cohort and the multifaceted nature of our analysis offer a relatively novel perspective. By leveraging a three‐wave longitudinal structure, we were able to derive several distinct conclusions—such as the divergent risk trajectories of BMI and WC—that refine our understanding beyond what is available in existing cross‐sectional literature.

Additionally, it is important to acknowledge that certain confounding biases persist, which can be challenging to fully mitigate. We had to face selection bias. During the participant enrollment phase, we regrettably had to exclude nearly 13,000 individuals due to the lack of blood and physical examination data, as well as information on knee pain. To rigorously address this, we conducted an IPTW sensitivity analysis. The weighted models revealed that the magnitude of the associations was virtually unchanged, with minimal attenuation observed. The fundamental associations remained statistically significant and highly consistent with our primary findings. This robust result firmly validates our conclusion. The absence of data regarding knee pain severity in the CHARLS database represents a major limitation. This omission meant that we were unable to evaluate whether a potential dose–response relationship exists between body shape indices and the severity of CKP. Besides, the CHARLS database lacks detailed records on histories of knee surgery. While we adjusted for a wide range of organic pathologies and analgesic use, the potential impact of previous orthopedic interventions on knee pain development could not be fully evaluated and should be addressed in future specialized clinical cohorts. Moreover, the CHARLS database primarily focuses on middle‐aged and older adults, who might have a biased comprehension of the questionnaire. Significantly, across each wave of the survey, there was a lack of detail regarding whether these participants had received treatment for knee pain, which could potentially have compromised the accuracy of our findings. Finally, the data analysis was conducted using the CHARLS database, which is confined to older adults residing in China, and it should be acknowledged that WC cutoff values vary significantly across ethnic groups. As our study focused exclusively on the Chinese population, the findings may have limited generalizability to other ethnicities. To extrapolate our findings to broader populations and enhance the validity of our conclusions, further validation through additional cohort studies is essential.

## 5. Conclusion

In this research, we delved into the association between obesity and CKP within a group of retired Chinese older adults. In short, abdominal obesity, BMI, and WC should be regarded as risk factors of CKP, whereas VAI, CI, and body weight should not be. BMI was nonlinearly related to CKP, whereas WC was linearly related. Notably, the clinical magnitude of the associations for BMI and WC remains modest compared to the robust risk conferred by abdominal obesity. We recommend that when devising public health policies aimed at addressing chronic CKP in obese populations, it is essential to prioritize the assessment of abdominal obesity alongside BMI.

**Nomenclature**
CKP: Chronic knee pain
SBP: Systolic blood pressure
DBP: Diastolic blood pressure
BMI: Body mass index
TG: Triglyceride
HDL‐C: High‐density lipoprotein cholesterol
WC: Waist circumference
VAI: Visceral adiposity index
CI: Conicity index

## Funding

This study was supported by the Shenzhen People’s Hospital Physician Scientist Training “Five Three Program” (SYWGSJCYJ202509).

## Ethics Statement

This study was a secondary analysis of data from the China Health and Retirement Longitudinal Study (CHARLS) and involved no new participant recruitment or primary data collection. Ethical approval for the original CHARLS data collection was granted by the Biomedical Ethics Review Board of Peking University (IRB00001052‐11015), and written informed consent was obtained from all participants.

## Conflicts of Interest

The authors declare no conflicts of interest.

## Supporting Information

Additional supporting information can be found online in the Supporting Information section.

## Supporting information


**Supporting Information 1** Table S1: Missing number for variables.

**Supporting Information 2** Table S2: Baseline characteristics of missing variables before multiple interpolation.

**Supporting Information 3** Table S3: Sensitivity analysis of the associations between obesity indices and incident CKP using IPTW.

## Data Availability

The data that support the findings of this study are available on request from the corresponding author. The data are not publicly available due to privacy or ethical restrictions.

## Appendix A

The analytical scripts and statistical code used in this study are available from the corresponding author upon reasonable request.
