# Supplementary material for: Body Shape Indices and Chronic Knee Pain in Chinese Aging Population: A Longitudinal Analysis of Abdominal Obesity, BMI, and Waist Circumference
Source: J Obes. 2026 Sep 28;2026:2379392. doi: 10.1155/jobe/2379392 (PMC13620403; doi:10.1155/jobe/2379392)
Supplement: Supplementary file 1 [file JOBE-2026-2379392-s001.docx]

TableS3 Sensitivity analysis of the associations between obesity indices and incident CKP using IPTW

| Variables | Logistic Model | | IPTW Adjusted Model | | ΔOR |
| --- | --- | --- | --- | --- | --- |
|  | OR（95%CI） | P-value | Weighted OR (95% CI) | P-value |  |
| BMI | 1.03 (1.01 ~ 1.05) | 0.020 | 1.03 (1.01 ~ 1.05) | 0.004 | 0.047% |
| WC | 1.01 (1.01 ~ 1.02) | 0.011 | 1.01 (1.00 ~ 1.02) | 0.008 | -0.128% |
| VAI | 1.01 (0.99 ~ 1.02) | 0.527 | 1.01 (0.99 ~ 1.03) | 0.311 | 0.074% |
| CI | 1.66 (0.90 ~ 3.05) | 0.104 | 1.82 (1.00 ~ 3.31) | 0.051 | 2.675% |
| Abdominal obesity | 1.36 (1.15 ~ 1.60) | <.001 | 1.35 (1.16 ~ 1.58) | <.001 | -1.172% |

Abbreviations: CKP: chronic knee pain; IPTW: inverse probability treatment weighting; BMI: body mass index; WC: waist circumference; VAI: visceral fat index; CI: conicity index
