# Supplementary material for: Body Shape Indices and Chronic Knee Pain in Chinese Aging Population: A Longitudinal Analysis of Abdominal Obesity, BMI, and Waist Circumference
Source: J Obes. 2026 Sep 28;2026:2379392. doi: 10.1155/jobe/2379392 (PMC13620403; doi:10.1155/jobe/2379392)
Supplement: Supplementary file 2 [file JOBE-2026-2379392-s002.docx]

**Table S2 Baseline Characteristics of missing variables before multiple interpolation.**

| Variables | Total | Participants without CKP | Participants with CKP | Statistic | *P* |
| --- | --- | --- | --- | --- | --- |
|  |  |  |  |  |  |
| SBP,mmHg | 126.91 ± 19.78 | 126.29 ± 19.81 | 128.59 ± 19.59 | t=-2.94 | **0.003** |
| DBP,mmHg | 74.88 ± 11.10 | 74.74 ± 11.05 | 75.26 ± 11.21 | t=-1.18 | 0.239 |
| Drinking status, n(%) |  |  |  | χ²=11.77 | **0.003** |
| No | 1979 (78.63) | 1390 (77.01) | 589 (82.72) |  |  |
| Less than once a month | 254 (10.09) | 203 (11.25) | 51 (7.16) |  |  |
| More than once a month | 284 (11.28) | 212 (11.75) | 72 (10.11) |  |  |
| Smoking status, n(%) |  |  |  | χ²=7.56 | **0.006** |
| No | 1075 (35.83) | 813 (37.31) | 262 (31.91) |  |  |
| Yes | 1925 (64.17) | 1366 (62.69) | 559 (68.09) |  |  |

Abbreviations: CKP: chronic keen pain; SBP: systolic blood pressure; DBP: diastolic blood pressure;

Notes: Continuous variables were expressed as mean±standard deviation (SD) in case of normal distribution and compared between two groups by t-test or Mann-Whitney test. If the count variable had a theoretical number<10, Fisher’s exact probability test was used. Categorical variables are presented as counts (percentages) and compared by Chi-square test

t
