# Supplementary material for: Body Shape Indices and Chronic Knee Pain in Chinese Aging Population: A Longitudinal Analysis of Abdominal Obesity, BMI, and Waist Circumference
Source: J Obes. 2026 Sep 28;2026:2379392. doi: 10.1155/jobe/2379392 (PMC13620403; doi:10.1155/jobe/2379392)
Supplement: Supplementary file 3 [file JOBE-2026-2379392-s003.docx]

**Table S1 Missing number for variables.**

| **Variables** | **Missing number (%)** |
| --- | --- |
| Age | 0 (0) |
| Gender | 0 (0) |
| Education level | 0 (0) |
| Current married | 0 (0) |
| Current residence | 0 (0) |
| Smoking | 288 (8.76%) |
| Drinking | 771 (23.45%) |
| SBP | 39 (1.19%) |
| DBP | 41 (1.25%) |

Abbreviations: SBP: systolic blood pressure; DBP: diastolic blood pressure;
